# Nurse and caregiver skill mix, staffing levels, and staff allocation in long-term care facilities: A scoping review

**DOI:** 10.4102/hsag.v31i0.3137

**Published:** 2026-06-29

**Authors:** Emerentia C. Nicholson, Justine C. Baron, Mariana M. van der Heever, Cornelle Young, Anita S. van der Merwe

**Affiliations:** 1 Department of Nursing and Midwifery, Faculty of Medicine and Health Sciences, Stellenbosch University, Cape Town, South Africa

**Keywords:** allocation, caregivers, long-term care, nurse staffing, skill mix

## Abstract

**Background:**

More older people require healthcare services, increasing the demand for nursing care. Long-term care facilities (LTCFs) are required to avail nurse staffing, a skill mix and allocation that is aligned with residents’ acuity and within prescribed legislation. However, facilities struggle to maintain the prescribed nurse staffing.

**Aim:**

A scoping review was conducted to map the available literature on implementing nurse and caregiver staffing models within LTCFs regarding skill mix, staffing levels, and staff allocation aligned with residents’ acuity.

**Setting:**

Studies conducted in formal long-term care settings for older people.

**Methods:**

The review followed the Joanna Briggs Institute’s methodological framework. The search included four databases: PubMed and MEDLINE, CINAHL, Cochrane Library–Wiley, and Sabinet African Journals, for studies published from 2010 to 2024. Trustworthiness was ensured by applying principles such as credibility, dependability, confirmability, and researcher reflexivity. The final sample comprised 20 studies.

**Results:**

Staffing levels for all categories of nurses and caregivers were often lower than recommended by experts. The number of registered nurses versus caregivers in the total staff mix was low, with caregivers providing most of the resident care. The use of acuity-adjusted staffing was not often reported.

**Conclusion:**

The appropriate type and number of staff in LTCFs are essential. In addition, allocating the correct type and number of staff according to residents’ acuity levels may improve resident outcomes.

**Contribution:**

This review may inform policymakers regarding the implementation of staffing models and the importance of aligning the allocation of tasks with the nurses’ scope of practice, the caregivers’ job descriptions, and the residents’ acuity.

## Introduction

The world’s population is ageing: 10% of people were older than 65 in 2022, and projections indicate an increase to 16% by 2050 (United Nations 2022). Consequently, the World Health Organization’s (WHO) Global Strategy and Action Plan on Ageing and Health urges countries to prioritise elderly care (WHO 2015). Older people tend to present with more signs of dementia, show functional decline, and are prone to comorbidities and chronic diseases (WHO 2015), leading to care dependencies. With an increase in their acuity, they thus need assistance with their activities of daily living, such as bathing (Hamel et al. 2021; Mlinac & Feng 2016), and professional assistance with medications and health conditions (Hamel et al. 2021). Older persons or their relatives may seek care in long-term care facilities (LTCFs) (Aboderin 2019). As more older people seek care in LTCFs, it may add pressure to the available resources in these facilities, including staffing, infrastructure, equipment, and supplies, which are necessary for the safe and comprehensive care of the elderly (Page et al. 2023). Staffing models, or plans, are used to determine the number of nurses and caregivers, the required skill mix, and the allocation of these staff to provide resident care. As a result, staffing models should help LTCFs, including those in low- and middle-income countries such as South Africa, to ensure that residents’ needs are met through appropriate staff allocation.

Most sub-Saharan countries have minimal or no regulations governing care for older people in formal settings (Dyer et al. 2019; WHO 2017). Sub-Saharan countries that have legislation governing formal elderly care include Zimbabwe (Madungwe, Mupfumira & Chindedza 2011), Tanzania (Van Eeuwijk 2014), and Namibia. However, besides South Africa, the researchers found no formalised LTCF staffing standards in sub-Saharan countries.

This scoping review was undertaken as part of a larger project to develop a framework to inform staffing models for LTCFs in resource-constrained contexts. Critical realism, as viewed by Roy Bhaskar, provided the philosophical foundation for the study (Bhaskar 2008). Bhaskar (2008) suggested that there are three nested forms of knowledge or truth, also known as domains. A researcher may observe truth or knowledge but must also delve deeper to uncover associations among role players, stakeholders, and underlying mechanisms. This deeper exploration may help researchers to understand the implementation of staffing models in LTCFs in certain settings (Bhaskar 2008). Furthermore, the study was underpinned by Mueller’s ‘Framework for Nurse Staffing in Long-term Care Facilities’ (Mueller 2000), which aims to guide nurse managers in observing residents to determine their staffing needs and in allocating staff to ensure quality care. The central concepts of a staffing model in Mueller’s framework were used in this study, such as skill mix, staffing levels, and staff allocation aligned with residents’ acuity.

### Skill mix

According to Mueller (2000), the skill mix within the team is one component to consider in a staffing model. Skill mix includes staff with assorted skills, qualifications, experience, proficiencies, and scope of practice (Backhaus et al. 2014). For example, a diverse skill mix may include nurses with degrees, newly qualified nurses, and caregivers who are not nurses. The skill mix is essential in LTCFs because the team needs higher levels of skill as residents become more frail (Boscart et al. 2018; Koopmans, Damen & Wagner 2018).

In South Africa, the skill mix includes registered nurses (RNs), comprising professional and general nurses (Republic of South Africa 2022), enrolled nurses (ENs), enrolled nurse assistants or auxiliary nurses (ENAs) (Republic of South Africa 2005), and caregivers (Republic of South Africa 2010a). Professional nurses hold 4-year bachelor’s degrees, provide comprehensive care by applying scientific principles across diverse service delivery contexts, and are allowed to manage the nursing department of an LTCF. General nurses have 3-year diplomas and provide general nursing care. General nurses, for example, promote public health, prevent disease, and address health problems by delivering nursing care to the overall population. When a healthcare unit operates within a larger facility, general nurses may manage a subunit but are not allowed to oversee the total nursing department of an LTCF (Republic of South Africa 2022). The ENs have 2 years of training and provide basic nursing care under RN supervision; however, this legacy qualification was phased out. The ENs with legacy qualifications remain part of the South African workforce and are therefore included in this review. The ENs are responsible, for example, for monitoring residents’ vital signs and reactions to medication, assisting with procedures, wound care, and hygiene (South African Nursing Council 1984). The ENAs have 1 year of training. A professional or general nurse delegates basic nursing care to ENAs. Basic nursing care includes assisting healthcare users with activities of daily living in accordance with prescribed standards of care, to promote and maintain their health status (Republic of South Africa 2022). A caregiver in South Africa includes anyone performing caregiving tasks in the formal sector but is not a qualified nurse (unlicensed staff) (Republic of South Africa 2006). Accredited training programmes are available for South African caregivers, such as the unit standard ‘Provide care to a frail person’. This programme comprises 12 credits, lasts 120 h, and is a National Qualification Framework (NQF) level 1 qualification. Alternatively, a longer community care programme is available at NQF levels 1, 2, or 3 (South African Qualifications Authority 2022). Caregivers assist residents with activities of daily living, including meal support, mobility, hygiene, and toileting (Republic of South Africa 2015).

There is a global shortage of health workers, and more than 50% of this shortfall comprises RNs and midwives (WHO 2022). This shortfall often leads to the replacement of RNs with nursing assistants to save costs (Shin & Hyun 2015). Substituting RNs with less-qualified staff may increase the number of less-qualified nurses in the skill mix, potentially leading to poorer resident outcomes (Harrington et al. 2020), while more RNs in the skill mix result in, for example, fewer urinary tract infections (Cho et al. 2020).

### Staffing levels

Staffing models should also include staffing levels, i.e. the quantity of caregivers and nurses (including all categories) available in the LTCF for resident care (Butler et al. 2019). Staffing levels are frequently expressed as staff-to-resident ratios, or as hours of care per resident day (HPRD). Authors found that residents’ activities of daily living improved with higher nurse staffing levels, resulting in lower hospitalisation rates (Harrington et al. 2020), and fewer adverse events and fewer deaths (Griffiths et al. 2018). Conversely, inadequate nurse staffing levels may compromise resident safety, lead to higher nurse workloads, burnout, and job dissatisfaction (Al-Jumaili & Doucette 2018; Griffiths et al. 2018).

### Staff allocation

According to Mueller (2000), staff allocation is another component to consider in a staffing model. Therefore, along with having sufficient nurses and caregivers and the right skill mix (different categories of nurses and caregivers), the LTCFs must assign staff in accordance with the residents’ acuity (Beckett et al. 2021; Butler et al. 2019), as the residents’ acuity determines the type of nursing care needed (Brennan & Daly 2009; Juvé-Udina et al. 2019). Long-term care facilities must also consider the legal parameters within which each nurse category may practice and which tasks they may perform (Republic of South Africa 2022), as determined by the country’s regulatory authority or statutory professional council, to ensure adherence to legislation. Caregivers’ job descriptions must comply with applicable legislation, and LTCFs must ensure caregivers are competent to perform assigned tasks.

The LTCFs face staffing deficiencies worldwide (Sato et al. 2017). These shortages lead to lower staffing levels and inadequate staff mixes that do not meet the recommended standards for LTCFs, especially regarding RNs (Brühl, Planer & Hagel 2018). With inadequate staffing and a diluted skill mix, LTCFs struggle to assign competent staff, especially when residents require higher levels of care (Estabrooks et al. 2020). Likewise, South African LTCFs also face nurse shortages and an inappropriate skill mix (Republic of South Africa 2010b).

The objective of the scoping review was to explore the implementation of nurse and caregiver staffing models in resource-rich and resource-constrained LTCF settings. The aim was to map the available literature on the implementation of LTCF nurse and caregiver staffing, focusing on skill mix, staffing levels, and staff allocation. The research question was: ‘What are the characteristics of staffing models implemented in resource-rich and resource-constrained contexts?’ The scoping review was guided by three sub-questions: ‘(1) How do LTCFs implement staffing models regarding staffing levels, skill mix, and allocation of tasks? (2) Is allocating tasks aligned with the nurses’ scope of practice and the caregivers’ job descriptions as described in the relevant country’s legislation? and (3) Is the allocation of staff aligned with the acuity of the residents?’

## Research methods and design

### Study design

This scoping review was conducted using the JBI methodological framework (Joanna Briggs Institute 2015) and the Peer Review of Electronic Search Strategies (PRESS) (eds. Aromataris et al. 2024). A priori protocol was developed to guide the scoping review (unpublished).

### Eligibility criteria

The eligibility criteria for selecting sources were based on the Participants, Concept, and Context (PCC) framework (Peters et al. 2020). Evidence was included if caregivers (or their counterparts in other countries) or any category of nurses, regardless of ethnicity, age, or gender, provided direct care to residents in LTCFs. Studies were considered if they contained at least two of the three central concepts identified by Mueller (2000), i.e. staffing levels, skill mix, and staff allocation according to residents’ acuity. Studies were included if they were conducted in formal long-term care settings for older people, thus, over 60 years old (WHO 2022), in either resource-rich or resource-constrained contexts. Studies within community settings (e.g. home-based care), studies conducted in languages other than English, studies for which the full articles were unavailable, and studies in which most data were collected before 2010 were excluded. The researchers decided to include data collected after 2010 because South Africa published its staffing model only in that year (Republic of South Africa 2010a) and to ensure the inclusion of more updated literature.

### Types of sources

Sources included primary research studies employing quantitative and qualitative methods, as well as grey literature (national and provincial legislation, acts, guidelines), policies, conference articles, dissertations, and theses. Scoping and systematic reviews were excluded because they contained synthesised evidence; however, Google Scholar was used to search their reference lists for additional articles.

### Search strategy

A qualified librarian assisted with developing a search strategy to identify relevant sources using Medical Subject Headings (MeSH) terms, Boolean operators, and the keywords in the abstract. Pilot screening was performed to ensure that the keywords produced appropriate results. After that, a three-step search process was followed (Peters et al. 2020), where the first-level limited search involved searching for text words in titles, abstracts, and indexed keyword lists in PubMed and MEDLINE. Table 1 provides an example of search strings for PubMed and MEDLINE.

**TABLE 1:** Example of search strings for PubMed and MEDLINE.

#	PubMed and MEDLINE search strings	Filters	# of results *n* = 446
1	‘long-term’ OR ‘nursing home’ AND ‘nurse staffing models’ OR ‘staffing models’ OR ‘staffing strategy’ NOT hospital	Full text, from 2010/1/1 to 2023/12/31	116
2	‘long-term’ OR ‘nursing home’ AND ‘nurse staffing levels’ OR ‘nurse staffing ratio’	Full text, from 2010/1/1 to 2023/12/31	53
3	‘staffing levels’ AND Nurse AND ‘long-term’	Full text, from 2010/1/1 to 2023/12/31	44
4	‘long-term’ OR ‘nursing home’ AND ‘skill mix’ OR ‘staffing mix’ OR ‘nurse skill mix’	Full text, from 2010/1/1 to 2023/12/31	115
5	‘long-term’ OR ‘nursing home’ AND ‘staff allocation’ OR ‘personnel allocation’ OR ‘nurse allocation’ OR ‘staff scheduling’	Full text, from 2010/1/1 to 2023/12/31	101
6	‘nurse staffing models’	Full text, from 2010/1/1 to 2023/12/31	17

*Source*: Adapted from Nicholson, E.C., Van der Heever, M.M., Young, C. & Van der Merwe, A., 2024, ‘Developing a framework to inform staffing models for long-term care facilities in resource-constrained contexts’, PhD thesis, Stellenbosch University, Stellenbosch #, number.

After the initial search, the strategy was adapted. Two reviewers independently used the same search strings to conduct a comprehensive search in PubMed and MEDLINE, CINAHL, Cochrane Library–Wiley, and Sabinet African Journals. The full search strings for all the databases are available as Online Appendix 1. Lastly, the researchers manually searched the reference lists of the selected studies for additional studies, which were then searched on Google Scholar. The original search took place in December 2022 and a final search was conducted in December 2024.

### Study selection

**FIGURE 1:**
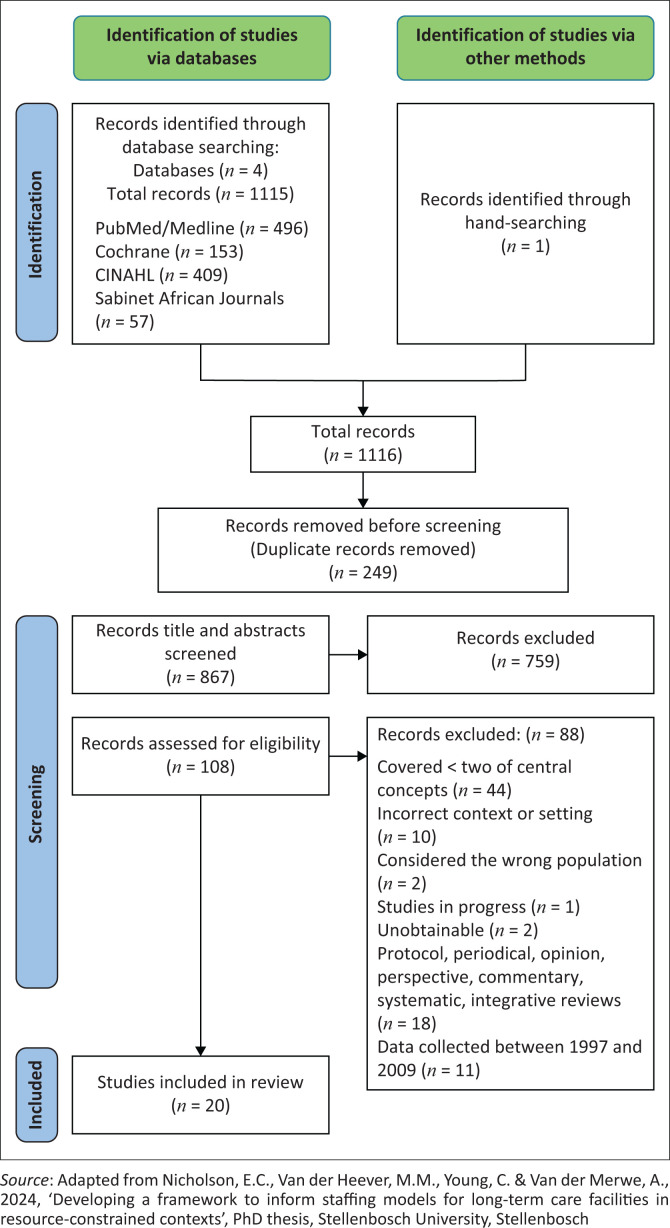
PRISMA flow chart of the literature identification and selection process.

JBI’s three-step process was used (Joanna Briggs Institute 2015). Two reviewers (ECB and JCB) independently screened the titles and abstracts manually against the inclusion criteria (Figure 1). After the initial title and abstract search, the two reviewers retrieved the full articles and conducted independent assessments. Discussions between the two reviewers resolved any disagreements about whether studies should be included or excluded from the review. A third reviewer (MMvdH) conducted a randomised assessment to verify the accuracy of the selection process and inclusion criteria.

### Data abstraction

A custom tool was used for data abstraction, and two reviewers then randomly tested it on three articles to revise it as needed (available as Online Appendix 2). The general information was recorded in a tabulated format (Joanna Briggs Institute 2015) and included the charting of authorship, publication year, country of origin, income level (high, medium, or low-income country), data sources used, study design, sample description and size, study aims, settings, and outcomes. The researchers did not critically appraise the included studies because the purpose of scoping reviews is to map available evidence irrespective of quality (Peters et al. 2020). Themes were used to organise the results.

### Trustworthiness

Trustworthiness was ensured through a detailed methodology description to promote transparency, enhance the credibility and reliability of the findings, and enable readers to evaluate the transferability of the scoping review to their own settings (Batten & Brackett 2021). A research question and sub-questions were developed, eligibility criteria were set, keywords were identified, and the search strategy was outlined. The inclusion of various electronic databases facilitated method triangulation (Batten & Brackett 2021). Gathering data from varied sources facilitated data triangulation (Lincoln & Guba 1985). The researchers practised reflexivity by considering their preconceived ideas and assumptions (Dwyer 2009), and carefully reflected on whether the data related to the research question and the study’s purpose, as well as with the concepts of staffing levels, skill mix, and staff allocation aligned with residents’ acuity, nurses’ scope of practice, and caregivers’ job descriptions.

### Ethical considerations

An application for full ethical approval was made to the Stellenbosch University’s Health Research Ethics Committee and ethics consent was received on 18 November 2022. The ethics approval number is S22/10/216.

## Results

### Study selection

Figure 1 contains a PRISMA flow chart to display the study selection and screening process, and reasons for excluding studies. Through four database searches, 1115 studies were identified. A manual search of the reference lists identified an additional study. The final sample included 20 studies.

### Study’s characteristics

Table 2 presents the characteristics of the studies. The studies were published between 2012 and 2022. All the data were collected in LTCFs in high-income countries. Data from low- and middle-income countries were unavailable. Nevertheless, resource constraints may also occur in LTCFs in higher-income countries, regardless of income level, making the results from these studies applicable. The studies included the United States (*n* = 8, 40%) (Chappell, Kirkham & Seitz 2022; Gorges & Konetzka 2020; Lerner 2013; Schnelle et al. 2016; Shin 2013; Shin, Park & Huh 2014; Yang et al. 2021; Yoon et al. 2022), South Korea (*n* = 6, 30%) (Kim & Han 2018; Lee et al. 2022; Shin 2018, 2019; Shin & Hyun 2015; Shin & Shin 2019), Canada (*n* = 1, 5%), (McCloskey et al. 2015) Germany (*n* = 2, 10%) (Zimmermann & Pfaff 2018; Zirves, Demirer & Pfaff 2021), and the Netherlands (*n* = 1, 5%) (Tuinman et al. 2016). One study collected data from the United States, Canada, England, Germany, Norway, and Sweden (*n* = 1, 5%) (Harrington et al. 2012), while another collected data from the United States and South Korea (*n* = 1, 5%) (Lee, Shin & Harrington 2015).

**TABLE 2:** Characteristics of the included studies.

Author(s), year published	Country, income level	Setting	Study design	Data sources	Description of sample	Sample size	Study purpose	Outcomes
Chappell et al. (2022)	United States (High-income country)	United States nursing homes with more than 50 beds, complete staffing data, and were under the federal government’s jurisdiction.	Quantitative study: Cross-sectional design	Nursing Home Compare LTCFocus datasets	RNs, LPNs, NAs, long-stay residents	*n* = 10 436 nursing homes	To investigate the relationship between staffing and prescribing antipsychotic medications in LTCFs between 2016 and 2018	When the total staffing levels were higher, antipsychotic medication prescribing rates were lower. When the licensed staff (RNs + LPNs) increased with 1 HPRD, inappropriate antipsychotic prescribing decreased by 3.09%. Nursing aide staffing was not associated with residents’ use of antipsychotic medication.
Gorges and Konetzka (2020)	United States (High-income country)	All United States nursing homes in the CMS COVID-19 Nursing Home Dataset	Quantitative study: Cross-sectional design	CMS Nursing Home Compare archives LTCFocus datasets	RNs, LPNs, CNAs, residents	*n* = 13 167 nursing homes	To understand the relationship between COVID-19 cases, the impact of outbreak severity, and baseline staffing	When the RN HPRD was higher, there was a higher possibility of experiencing COVID-19 cases (the reason was not provided) but lower odds of COVID-19 outbreaks (because of efforts to contain transmission). Low LPN hours were unrelated to COVID-19 outbreaks, but fewer deaths were reported. When the nursing aide levels were higher, the odds of COVID-19 outbreaks were lower, and there were fewer deaths.
Harrington et al. (2012)	United States Canada England Germany Norway Sweden (High-income countries)	Nursing homes in the United States, Canada, England, Germany, Norway, and Sweden	Quantitative study: Cross-sectional design	Staffing regulations Policies Statistical data from government staffing reports and documents	RNs, LPNs, CNAs, care workers	Data (number not specified) from documents, regulations, internet searches, government websites, research articles and reports	To collect data to compare nurse staffing standards and levels across six countries: the United States, Canada, England, Germany, Norway, and Sweden	Staffing standards and actual staffing levels were lower in most countries than what an expert panel and the CMS in the United States recommend. Most countries do not adjust staffing standards to residents’ acuity. Actual staffing data in nursing homes were sometimes unavailable, and the care models differed.
Kim and Han (2018)	South Korea (High-income country)	All long-term care hospitals (nursing homes) in South Korea	Quantitative study: Longitudinal design	HIRA website	RNs, CNAs, residents	Long-term care hospitals (South Korean nursing homes): *n* = 481 in 2010 *n* = 631 in 2012 *n* = 881 in 2013	To examine hospitals that provided long-term care from 2010 to 2013 and explore the link between patient outcomes and staff turnover during that period	Resident acuity increased in the LTCFs, leading to higher care needs, which necessitated staff with higher educational preparation. However, the skill mix in the LTCFs decreased from 2010 to 2013. Consequently, the RN-to-patient ratio decreased, leading to adverse outcomes.
Lee et al. (2015)	South Korea United States (High-income countries)	Nursing homes	Quantitative study: Descriptive design	Documents and regulations of nursing home staffing standards from government websites, government documents, research articles, reports	RNs, LPNs, CNAs, caregivers	–Data from *n* = 50 US states and the District of Columbia in 2010 –Medicare Nursing Home Compare Website 2013 –Data about each country’s nomenclature for nursing homes, number of homes, educational and training requirements	To compare staffing standards and nurse levels and education and training requirements in United States and South Korean nursing homes	Korean RN education requirements compared with those of United States RNs; Korean CNAs’ job descriptions compared with those of United States LPNs; and Korean care workers’ job descriptions compared with those of United States CNAs. The United States appeared to have higher RN and CNA standards and levels but lower caregiver levels than South Korea. South Korean LTCFs were required to have higher staffing levels when residents’ acuity levels increased, while the United States did not apply acuity-based staffing.
Lee et al. (2022)	South Korea (High-income country)	All nursing homes in South Korea	Quantitative study: Descriptive design	Korean Government’s Information Disclosure System Korean National Health Insurance Corporation website	RNs, CNAs, residents	*n* = 3389 nursing homes *n* = 118 315 residents	To examine organisational factors and staffing characteristics in nursing homes during the peak of the COVID-19 pandemic. The authors explored the relationship between staff-to-resident ratios, RN proportions, and the number of resident deaths.	Nursing homes that maintained a significant proportion of RNs within their nursing staff, resulting in a lower RN-to-resident ratio (1:113), experienced no COVID-19 outbreaks. Conversely, nursing homes with a higher RN-to-resident ratio (1:132) did encounter COVID-19 outbreaks. When compared to nursing homes without COVID-19, which had a CNA-to-resident ratio of 1:21, those with COVID-19 outbreaks had a slightly higher CNA-to-resident ratio of 1:19. The care worker-to-resident ratios were the same (1:2) between nursing homes with or without COVID-19 outbreaks. However, the care worker-to-resident ratios (1:2) were positively correlated with the infection rate (*p* = 0.33).
Lerner (2013)	United States (High-income country)	Nursing homes in the state of Maryland	Quantitative study: Cross-sectional design	Data from the Nursing Home Compare website	RNs, CNAs	*n* = 225 nursing homes	To explore whether nurse staffing levels and skill mix influenced Maryland nursing facilities’ deficiencies in terms of severity and numbers	Having more RNs than CNAs in the skill mix was linked to lower deficiency severity, although not with the number of deficiencies. Higher CNA HPRD was associated with fewer deficiencies being issued.
McCloskey et al. (2015)	Canada (High-income country)	Not-for-profit nursing homes in one Canadian province	Quantitative study: Cross-sectional design	Primary data: observations	RNs, LPNs, resident attendants and/or care aides	*n* = 7 nursing homes	To determine how nurses and caregivers spend their time in nursing homes and explored whether differences existed between the seven nursing homes, the work shifts, and the staff’s licensure levels	The seven included nursing homes all showed adherence to the government-mandated skill mix. RNs and LPNs performed activities that could have been delegated to non-regulated care workers. Care aides spent considerably more time on valueless tasks (e.g. searching for equipment) than other care providers, indicating poor efficiency.
Schnelle et al. (2016)	United States (High-income country)	Nursing homes nationwide in the United States	Quantitative study: Correlational design	CMS data	Nursing aides	*n* = 211 424 quarters of CMS data *n* = 13 533 nursing homes	To describe associations between nurse aide staffing levels and the activities of daily living (ADL) workload	The link between residents’ required care based on their ADL needs and staffing levels was weak. Nurse aides provide the most labour-intensive care to residents by assisting with ADLs, such as bathing, feeding, incontinence care, and mobility.
Shin (2013)	United States (High-income country)	One was government-owned, and there were 12 for-profit and 12 not-for-profit nursing homes in Iowa, United States. Rural nursing homes: 15, urban areas: 10	Quantitative study: Correlational design	OSCAR MDS version 3.0 (The Quality-of-Life section in MDS 3.0-questionnaire for residents)	Residents, RNs, LPNs/LVNs, CNAs	*n* = 231 residents *n* = 25 nursing homes	To investigate associations between nurse staffing and United States nursing home residents’ quality of life	A higher proportion of RNs to LPNs/LVNs and CNAs was associated with a higher ability of residents to function and complete tasks. However, as the proportion of RNs to LPNs/LVNs increased, it did not positively contribute to residents’ autonomy (possibly because the RNs are less involved in direct care activities such as clothing the residents) and spiritual wellbeing (maybe because RNs do not transport residents to religious services).
Shin (2018)	South Korea (High-income country)	South Korean nursing homes	Quantitative study: Longitudinal design	Primary data: Quarterly surveys	Nursing home administrators, RNs, and CNAs are workers	*n* = 45 nursing homes	To examine the longitudinal link between residents’ care quality and the available nurses	In a skill mix where more RNs and fewer CNAs or care workers were employed, residents were less aggressive, showed fewer signs of depression and weight loss, and were less on bed rest.
Shin (2019)	South Korea (High-income country)	South Korean nursing homes	Quantitative study: Longitudinal design	Six quarterly nurse staffing HPRD data and 15 quality care indicators	RNs, CNAs, care workers	*n* = 45 nursing homes	To develop appropriate optimisation models for determining the optimal nursing staff HPRD to achieve better care outcomes for nursing home residents	An increase of 12% in RN HPRD corresponded to a 3% enhancement in care quality. Similarly, a 20% increase in RN HPRD showed a 5% to 8% improvement in quality-of-care outcomes without increasing the CNA HPRD. Furthermore, a 30% increase in RN HPRD without an increase in CNA HPRD led to a 5% – 8% increase in the quality of all the care outcomes combined.
Shin and Hyun (2015)	South Korea (High-income country)	Nursing homes from six provinces in South Korea	Quantitative study: Cross-sectional design	Primary data: surveys	RNs, CNAs, care workers	*n* = 19 nursing homes	To investigate the link between nursing home quality of care and nurse staffing in Korea, using 15 quality-of-care indicators	Compared to certified nurse assistants, RNs contribute uniquely to resident outcomes in fall prevention, decreased aggressive behaviours, less use of tubes for feeding, and better mobility in nursing homes in South Korea. There was no significant statistical difference between the skill mix ratio of certified nurse assistants and qualified care workers in proportion to the total staffing and the quality-of-care outcomes.
Shin, Park and Huh (2014)	United States (High-income country)	Nursing homes within 100 miles of Buffalo, New York, United States.	Quantitative study: Cross-sectional design	OSCAR data Primary data using the Nursing Personnel Data Collection Tool Self-reported Quality of Life instrument	Residents, RNs, LPNs, CNAs	*n* = 142 residents *n* = 8 nursing homes	To explore associations between QOL and nurse staffing in Western New York State nursing homes	A higher portion of LPNs and CNAs than RNs in the total skill mix had a better impact on residents’ meaningful activities, security, and food enjoyment domains. In contrast, the more stable the RN staffing was (less turnover), the more content the residents were with their meaningful activities, security, and food enjoyment.
Shin and Shin (2019)	South Korea (High-income country)	Nursing homes were randomly sampled from 17 administrative districts in Korea.	Quantitative study: Cross-sectional design	Data from the 60 nursing homes and open-access government data	RNs, CNAs, care workers	*n* = 60 nursing homes	To explore links between care quality outcomes and nurse staffing by considering the healthcare market characteristics in South Korea	An increase in RN HPRD reduced the administration of psychotropic medications to residents, lowered the likelihood of residents experiencing inadequate nutrition and weight loss, and did not seem to have a significant effect on staff turnover.
Tuinman et al. (2016)	The Netherlands (High-income country)	Three chains of residential care facilities and nursing homes were selected in the north of the Netherlands. Five facilities were included: four residential care units, three somatic units, and six psycho-geriatric units.	Quantitative study: Cross-sectional design	Primary data: Structured questionnaires for observations Delphi panel	Residents, healthcare assistants, nursing assistants, RNs, and primary caregivers	*n* = 5 facilities that provided long-term care *n* = 19 RNs *n* = 89 nursing assistants *n* = 9 primary caregivers *n* = 19 healthcare assistants *n* = 335 residents	To examine relationships between how nurses use their time, the type of nursing staff, the acuity levels of residents, and the types of units by using standardised nursing intervention classification	There was no significant association between the time nurses spent on nursing intervention classification domains and residents’ acuity levels. Residents seemed to receive the same care regardless of their individual needs or acuity levels. Role differentiation appeared blurred because task allocation between RNs, nursing assistants, and primary caregivers was limited.
Yang et al. (2021)	United States (High-income country)	Medicare/ Medicaid-licensed nursing homes in the 2018 United States CASPER period.	Quantitative study: Cross-sectional design	CMS CASPER AHRF	RNs, LPNs, CNAs	*n* = 14 325 nursing homes	To examine nurse staffing levels and skill mix patterns and to determine whether these patterns affected rehospitalisation rates or recurrent emergency department visits	Nursing homes with a more significant proportion of RNs than the national average had the lowest emergency department visit and rehospitalisation rates. In contrast, the nursing homes that relied more on LPNs than RNs and CNAs for care delivery had the highest emergency department visits and rehospitalisation rates. A poor alignment existed between LPNs’ scope of practice and residents’ needs.
Yoon et al. (2022)	United States (High-income country)	Federal regulations govern United States nursing homes with Medicare and/or Medicaide licensure.	Quantitative study: Cross-sectional design	CASPER Payroll-Based Journal data	RNs, LPNs, CNAs, nurse aides in training, certified medication aides or technicians	*n* = 13 614 nursing homes	To explore the links between how often nursing homes received deficiency citations for improper psychotropic medication use in treating residents with dementia symptoms and nurse staff levels	Higher nurse and caregiver staffing levels in nursing homes were associated with fewer chances of receiving deficiency citations for improper use of psychotropic medications than in nursing homes with lower staffing levels.
Zimmermann and Pfaff (2018)	Germany (High-income country)	Most nursing homes were non-profit organisations in the federal states of North Rhine-Westphalia and Bavaria. The areas included Saarland, Schleswig-Holstein, Baden-Wuerttemberg, Rhineland-Palatinate, and Hesse.	Quantitative study: Correlational design	Project EQisA. data (Project EQisA was developed to examine quality outcomes in German nursing homes).	Residents, RNs, nursing assistants, additional care staff	*n* = 166 nursing homes *n* = 8665 residents	To determine the staffing level differences between facilities with residents who showed no weight loss and facilities with residents experiencing unintentional weight loss	Staffing levels varied between regions in Germany and were calculated based on the residents’ dependency levels. In weight loss, the nurse-resident ratio or staffing level seemed to be a factor for cognitively abled residents but did not affect those with cognitive impairment. RNs are responsible for residents’ nutrition, and additional care staff assist with eating. Nutritional care may improve with feeding assistance from the additional care staff.
Zirves et al. (2021)	Germany (High-income country)	Nursing homes from the Diocesan Caritas Association North Rhine-Westphalia	Quantitative study: Correlational design	Project in QS (Project used indicators to promote quality)	Residents, RNs, nursing assistants, additional care staff	*n* = 30 nursing homes, *n* = 1782 residents older than 80 years	To explore whether there was a link between residents older than 80, with or without dementia, and RN-to-resident ratios	Residents’ ability to organise their lives independently and maintain social contact when they did not have dementia was linked to the number of available RNs. This might have been because RNs can promote residents’ independence. However, the RN-to-resident ratio was not related to residents with dementia’s ability to organise their lives independently and maintain social contact. This may be because of RNs not being much involved with direct resident care and the residents with dementia already being restricted regarding their abilities.

*Source*: Adapted from Nicholson, E.C., Van der Heever, M.M., Young, C. & Van der Merwe, A., 2024, ‘Developing a framework to inform staffing models for long-term care facilities in resource-constrained contexts’, PhD thesis, Stellenbosch University, Stellenbosch

Note: Details of the cited articles are available in the full reference list of this manuscript.

AHRF, Area Health Resources File; CASPER, Certification and Survey Provider Enhanced Reporting System; CMS, Centres for Medicare & Medicaid Services; CNA, certified nurse assistant; DES, Discrete event simulation model; HIRA, Health Insurance Review and Assessment Service; HPRD, hours per resident day; LPNs, licensed practical nurses; LTCFocus, long-term care focus; LVNs, licensed vocational nurses; MDS, minimum data set; NAs, nursing assistants; OSCAR, Open Super-large Crawled Aggregated coRpus; RNs, registered nurses; LTCF, Long-term care facilities; US, United States.

Eleven studies (*n* = 11, 55%) used cross-sectional designs (Chappell et al. 2022; Gorges & Konetzka 2020; Harrington et al. 2012; Lerner 2013; McCloskey et al. 2015; Shin 2018; Shin et al. 2014; Shin & Shin 2019; Tuinman et al. 2016; Yang et al. 2021; Yoon et al. 2022), four studies (*n* = 4, 20%) used correlational designs (Schnelle et al. 2016; Shin 2013; Zimmermann & Pfaff 2018; Zirves et al. 2021), three studies (*n* = 3, 15%) were longitudinal (Kim & Han 2018; Shin 2018, 2019), and two studies (*n* = 2, 10%) were descriptive (Lee et al. 2015, 2022). Populations included RNs, licensed practical or vocational nurses (LPNs or LVNs), nurse assistants (NAs), certified nursing assistants (CNAs), care workers, and residents. Most studies (*n* = 15, 75%) collected secondary data from government staffing websites, payroll-based journal data, medical insurance websites, government documents, research articles, reports, and quarterly data from nursing homes (Chappell et al. 2022; Gorges & Konetzka 2020; Harrington et al. 2012; Kim & Han 2018; Lee et al. 2015, 2022; Lerner 2013; Schnelle et al. 2016; Shin 2013, 2019; Shin & Shin 2019; Yang et al. 2021; Yoon et al. 2022; Zimmermann & Pfaff 2018; Zirves et al. 2021). Five studies (*n* = 5, 25%) collected primary data (McCloskey et al. 2015; Shin 2018; Shin & Hyun 2015; Shin et al. 2014; Tuinman et al. 2016).

### Synthesis of results

The synthesised results answered the first research question: ‘How do LTCFs implement staffing models regarding skill mix, staffing levels, and task allocation according to residents’ acuity?’

### Skill mix

Countries’ national staffing standards varied regarding their skill mix (Harrington et al. 2012; Lee et al. 2015; McCloskey et al. 2015; Shin 2019; Shin & Shin 2019; Zimmermann & Pfaff 2018; Zirves et al. 2021). According to studies conducted in 2018 and 2021, Germany required that half of the care staff must be RNs, spread over 24 h but had no additional specific requirements for the rest of the staff (Zimmermann & Pfaff 2018; Zirves et al. 2021). Norway, Sweden, and England did not specify staffing standards (Harrington et al. 2012). In 2019, South Korea required one RN or CNA per 25 residents for nursing homes exceeding 30 beds, and one RN or CNA for nursing homes with fewer than 10 beds and up to 30 beds. The remaining staff comprised care workers (Shin 2019; Shin & Shin 2019). Studies conducted between 2012 and 2015 reported that federal United States standards required one RN to be on duty for 8 h every weekday, with an RN and an LPN covering the remaining two 8-h shifts. A director of nursing must be full-time employed, and there must be sufficient staff to ensure residents’ well-being. The different states within the country may set standards that exceed the federal government’s staffing standards (Harrington et al. 2012; Lee et al. 2015).

Authors reported in 2012 and 2015 that Canada’s different provincial governments determined their staffing standards (Harrington et al. 2012; McCloskey et al. 2015).

In the studies, the proportion of RNs among the total nurse and caregiver staff ranged from 13.2% (Yang et al. 2021) to 56.6% (Zimmermann & Pfaff 2018). In South Korea, an RN or CNA may be used interchangeably, with the proportion of RNs or CNAs indicated in the study as 15.87% (Lee et al. 2022). Yang et al. (2021) indicated in 2021 the proportion of LPNs in their United States study as 23.4%, while McCloskey et al. (2015) in Canada in 2015 indicated 40%. The proportion of caregivers ranged from 40% in Canada (McCloskey et al. 2015) to 84.13% in South Korea (Lee et al. 2022).

Registered nurses undergo between 3 and 4 years of training (Zirves et al. 2021). Compared to other nurse categories and caregivers, RNs use higher levels of clinical judgement, decision-making, and critical thinking when providing care (Chappell et al. 2022). In the United States, the category of LPN completes a 1-year full-time nursing course (Yang et al. 2021). South Korean CNA category’s job description seems comparable to LPNs in the United States (Lee et al. 2022). Certified nursing assistants in South Korea do not train at nursing colleges but attend private nursing institutes or occupational high schools (Shin 2019). Care workers in South Korea receive 240 h of home care training (Shin 2018, 2019) and have job scopes comparable to those of the United States CNAs (Lee et al. 2022). Unlicensed staff, such as care workers without nursing qualifications, are often used (Shin 2018, 2019).

### Staffing levels

Staffing levels, i.e. the number of staff required to provide care HPRD, also varied between provinces and countries. In 2011, the HPRD was 1.9 in Alberta, Canada, and 2.0 in Saskatchewan, Canada. The HPRD in Florida was 3.9 and 2.56 in South Carolina in the United States (Harrington et al. 2012). In Germany, residents highly dependent on care require ≥5 HPRD for assistance or ≥4 HPRD for basic care (Zimmermann & Pfaff 2018). In South Korea, national standards stipulate that for every 25 residents, there must be one CNA *or* RN, and that the care worker-to-resident ratio be maintained at 1:2.5 (Lee et al. 2022; Shin 2019). England’s 2011 regulations did not specify LTCF staffing levels; however, the registration authority set those levels based on residents’ needs (Harrington et al. 2012).

Chappell et al. (2022) conducted a cross-sectional study and found that across 10 436 United States LTCFs, the mean staffing level for RNs, LPNs, and nursing assistants was 3.69 HPRD. Harrington et al. (2012) compared staffing standards across countries in 2022, with the HPRD values as follows: United States: 3.9 HPRD, Canada: between 2.1 and 3.3 HPRD, England: 4.26 HPRD, and Sweden, with the highest staffing levels at 5.19 HPRD. A comparison of nurse staffing between South Korea and the United States showed that the United States RNs provided an HPRD of 0,63, versus 0,47 for RNs or CNAs in South Korea (Lee et al. 2015). In a study conducted across seven Canadian LTCFs, the staffing level averaged 3.1 HPRD (McCloskey et al. 2015).

Higher total staffing levels seemed to be linked to lower prescription rates of antipsychotic medications, better ability of staff to address residents’ behavioural and psychological symptoms (Chappell et al. 2022), and to allow RNs more time to explore possible causes of disruptive behaviours (Yoon et al. 2022). The probability that an LTCF had only one COVID-19 case was not affected by the number of staff, although more staff were associated with better COVID-19 case control (Gorges & Konetzka 2020). Lee et al. (2022) further found that a higher care workers-to-residents ratio was associated with more infections and higher mortality rates; thus, when more care workers were employed, but fewer RNs, infections and mortality rates were higher. Low staffing levels were associated with high workloads (Yoon et al. 2022) and the inability of the staff to assist residents with their daily living activities (Schnelle et al. 2016).

Four studies described the effect of increased staffing levels on resident outcomes. A 1-h increase in overall staffing HPRD led to a 0.75% reduction in the improper prescribing of antipsychotic medication. When the RNs’ HPRD was increased by 1 h, it led to a decrease of 2.25% in the improper prescribing of antipsychotic medications (Chappell et al. 2022), a 5.72% decrease in residents with bed rest (Shin 2018), and 6.8% fewer resident falls (Shin & Hyun 2015). A 12% increase in RN HPRD resulted in a 3% improvement in quality of care, while a 20% increase in RN HPRD led to a 5% – 8% improvement in quality of care (Shin 2019). A 1-h increase in LPN HPRD decreased inappropriate antipsychotic medicine prescribing by 1.83% (Chappell et al. 2022). However, when increasing the HPRD of CNAs and care workers by 1 h, aggressive behaviours in residents increased by 4.238%, residents on bed rest increased by 5.047% (Shin 2018), and there was a 5.3% increase in feeding tube use (Shin & Hyun 2015).

Only three studies reported a link between skill mix, staffing levels, and organisational outcomes. In Lerner’s (2013) study in 2013, 225 United States LTCFs with a mean total staffing of 1.71 HPRD received 9.76 deficiency citations for violating federal regulatory care standards. While some LTCFs received no citations, others received up to 51 deficiency citations (Lerner 2013). Similarly, Yoon et al. (2022) found that the likelihood of receiving deficiency citations was lower when staffing levels were 3.61 HPRD rather than 3.51 HPRD. In addition, low staff turnover leads to better quality of care, whereas high RN turnover worsens residents’ outcomes.

### Staff allocation

Staff allocation and the use of nurses and caregivers showed similarities across the countries. The roles and responsibilities of the RNs include taking responsibility for the overall nursing process (Zimmermann & Pfaff 2018), being accountable for evaluating overall nursing outcomes (Yoon et al. 2022), providing health education, conducting residents’ physical assessments (Lee et al. 2022), and overseeing nursing assistants and additional care staff (Zimmermann & Pfaff 2018). Registered nurses also tended to care for residents requiring higher levels of care (Tuinman et al. 2016). Between 3.5% and 28.9% of RNs’ time is spent on non-value-added resident-related activities, such as refilling stock items, searching for equipment and stock, issuing linen, and locating other staff. Furthermore, RNs spend about 16.9% of their duty time on walking through the unit and 41.7% on indirect resident care, including documentation and communication (McCloskey et al. 2015).

Authors found a lack of clarity between the roles of nurse categories and caregivers (Lee et al. 2022; McCloskey et al. 2015; Shin 2019; Shin & Hyun 2015; Shin & Shin 2019; Yang et al. 2021; Yoon et al. 2022). Licensed practical nurses appeared to work beyond their scope of practice when RNs were unavailable, but below their scope of practice when CNA shortages occurred (Yoon et al. 2022). In addition, poor role clarity led to RNs and LPNs performing various tasks that could have been delegated to unlicensed care workers (McCloskey et al. 2015). Care workers provide most of the direct resident care under the supervision of RNs and LPNs (Schnelle et al. 2016; Shin 2019; Yang et al. 2021; Zirves et al. 2021).

Acuity-based staffing is not commonly used in most countries, including the United States and Canada, which provided an answer to this scoping review’s third question: ‘Is the allocation of staff aligned to the acuity of the individual residents?’ Harrington et al. (2012) stated that not implementing acuity-based staffing standards may be the reason for the inability to address residents’ higher-level care needs. Germany may be an exception, as residents’ dependency levels informed staffing decisions (Zimmermann & Pfaff 2018). Care omissions were about 22% when residents’ acuity levels were high (Schnelle et al. 2016). However, the authors found that residents received similar care despite differing needs (Tuinman et al. 2016).

## Discussion

The scoping review mapped the results of 20 studies to present a comprehensive overview of the evidence available on how LTCFs implement staffing models regarding staffing levels, skill mix, and task allocation aligned with the nurses’ scope of practice, caregivers’ job descriptions, and residents’ acuity.

### Skill mix

The skill mix standards varied across and within countries, from no specific set standards to prescribing explicit staffing standards. South Africa also has explicit mandatory staffing standards, irrespective of province, whether urban or rural, and whether LTCFs were private or state-subsidised (Republic of South Africa 2010a). The RN numbers in the overall nurse and caregiver staffing in the studies appear low due to some countries’ standards, such as South Korea, where CNAs may be used instead of RNs (Cho et al. 2020; Shin et al. 2021). In contrast to the proportion of nurses in the skill mix in the included studies, the proportion of unlicensed caregivers in the total staffing was as high as 84.13% (Lee et al. 2022), despite LTCF residents requiring higher levels of (Bae & Kim 2020; Kim & Han 2018; Tuinman et al. 2016). South African staffing standards for frail residents require that 33% of the total staff be RNs, but 50% of the total RNs may be replaced by ENs, resulting in 16.5% RNs and 16.5% ENs. The remaining 66% of the staff may be ENAs, but 50% of the total ENAs may be replaced by caregivers, thus 33% ENAs and 33% caregivers (Republic of South Africa 2010a).

Authors found that more highly educated healthcare professionals are needed (Kim & Han 2018) because higher-qualified staff can better address residents’ higher care needs, thereby improving resident outcomes (Chappell et al. 2022). Overall, the quality of resident care is improved, with lower infection and mortality rates when more RNs are employed rather than care workers (Lee et al. 2022), leading to better quality of life for residents, less decline in residents’ functional abilities (Shin et al. 2021), and fewer pressure ulcers (Clemens et al. 2021; Jutkowitz et al. 2023; Shin et al. 2021). Thus, before determining the skill mix needed, LTCFs should identify which educational levels and skills are required to meet residents’ dependency levels and care needs (Harrington et al. 2012). In South African LTCFs, it may be more viable for financially struggling facilities to not only appoint professional nurses but also make greater use of general nurses (diploma-qualified). However, LTCFs should analyse gaps in general nurses’ skills and address them through in-service training. Although RNs are typically employed at a higher cost than LPNs or CNAs (or ENs and ENAs in South Africa), using RNs offers potential cost-effectiveness in contrast to applying a low-cost nurse staffing model. Investing in more RNs may improve the quality of care, result in fewer deficiency citations (Harrington et al. 2020; Perruchoud et al. 2021), and reduce costs associated with adverse events (Cho et al. 2020; Mukamel et al. 2023).

### Staffing levels

Studies have shown that staffing levels in LTCFs vary across countries, with 2.3 HPRD in the United States (Schnelle et al. 2016) and 5.19 HPRD in Sweden (Harrington et al. 2012). United States experts have suggested that LTCFs provide 4.55 HPRD, although many countries provide lower HPRD (Harrington et al. 2012). A minimum of 2.57 HPRD is prescribed for frail residents in South Africa (Republic of South Africa 2010a). Staff burnout may follow when staffing levels are low, as higher workloads increase stress, impede staff’s ability to assist residents with daily activities, and may lead to inadequate care (Perruchoud et al. 2021; Schnelle et al. 2016). Adverse events, such as increased use of psychotropic medications in residents, may occur when RN staffing levels are too low. Conversely, higher RN staffing can contribute to positive resident outcomes. Higher RN hours may help address residents’ behavioural issues when RNs have enough time (Yoon et al. 2022), thereby reducing the need for antipsychotic medications (Chappell et al. 2022). Fewer resident deaths were associated with higher staffing levels (Cho et al. 2020), emergency department visits and rehospitalisations were less (Yang et al. 2021), fewer residents were on bed rest, and there was less need to restrain residents (Shin 2018). Long-term care facilities can incur higher costs when they are required to use agency staff to fill staffing gaps, rather than increase baseline staffing levels above the minimum mandated standards (Griffiths et al. 2021).

### Staff allocation

Despite evidence that residents’ needs are increasing, most LTCFs did not use acuity-based staffing methods at the time of completing this review (Chappell et al. 2022; Zimmermann & Pfaff 2018). Authors found that residents receive similar care despite differing needs (Tuinman et al. 2016). Moreover, care omissions were higher when residents’ acuity was higher (Schnelle et al. 2016). Thus, before staff planning, LTCFs should assess residents’ needs, consider their acuity levels, and align staff’s educational levels, skills, and experience with those needs to ensure residents receive appropriate care (Harrington et al. 2012; Tuinman et al. 2016).

Despite the clear scope of practices for nurses, there is an increased lack of clarity between the roles of different nurse categories and caregivers (Lee et al. 2022; McCloskey et al. 2015). To reduce costs, LTCFs appear to rely on caregivers and LPNs rather than RNs. Consequently, less-qualified staff may assume responsibilities beyond their qualifications. Lee et al. (2022) found that 22% of South Korean LTCFs did not employ RNs but instead used CNAs, even though RNs and CNAs have different scopes of practice and qualifications (Lee et al. 2022; Perruchoud et al. 2021). Likewise, RN shortages may lead LPNs to work beyond their scope of practice, risking overextension (Yang et al. 2021). Despite the RNs’ and LPNs’ training and skill set, the results indicated that they perform activities that could have been delegated to lower-qualified staff (McCloskey et al. 2015). Registered nurses spend significant time on indirect resident care activities, such as walking through the units, reviewing documents, and recordkeeping (McCloskey et al. 2015; Tuinman et al. 2016).

However, these non-direct resident activities should support residents’ care. Registered nurses should review workflow processes and feel comfortable assigning tasks to care workers when those tasks do not benefit residents.

Consequently, RNs’ efficiency could be enhanced, and they could concentrate on completing tasks within the full scope of their practice (McCloskey et al. 2015). Furthermore, despite being unlicensed, caregivers provide most of the direct resident care and carry significant responsibilities (Lee et al. 2022; Yang et al. 2021). Thus, role clarifications are needed to ensure that tasks are assigned to the appropriate staff, enabling resource optimisation and facilitating quality care (McCloskey et al. 2015). In South Africa, policies that comply with the legal boundaries of caregivers’ job scope may prevent undesirable task-shifting from higher- to lower-qualified staff.

This scoping review confirmed that adequate staffing is viewed differently between countries and that staffing involves more than only having enough staff with the right qualifications. The review also highlights the importance of aligning the allocation of tasks with the nurses’ scope of practice, the caregivers’ job descriptions, and the acuity of the residents.

### Strengths and limitations of this review

During this scoping review, insights were gained into nurse and caregiver staffing in LTCFs. Developing a protocol to delineate the scoping review plan strengthened the review. Transparency was enhanced by using two independent reviewers to conduct a search strategy and document all the phases of the review process. A limitation of the scoping review was a lack of evidence from low- to middle-income countries. This may provide an inaccurate or biased perspective of the current state of skill mix, staffing levels, and staff allocation practices in LTCFs. Limiting the search to English-language studies from 2010 may have narrowed the scope of the review, potentially excluding relevant articles and introducing publication bias.

### Recommendations for future research

Further research is needed on nurse and caregiver staffing, especially in LTCFs in low- and middle-income countries. Given that caregivers provided most of the care, it is necessary to examine whether staffing deficiencies were associated with adverse events. In addition, research is needed to determine whether residents and family members are comfortable receiving most of their care from caregivers rather than from higher-qualified nurses.

## Conclusions

Older person care is a global concern that demands careful planning and the strategic use of resources. As older people increasingly require more care, it can place additional burdens on limited resources, including staff. Despite limited resources, LTCFs must ensure they have sufficient staff and a skill mix that meets legislative requirements and residents’ needs. Furthermore, aligning task allocation with nurses’ scope of practice, caregivers’ job descriptions, and residents’ acuity levels may improve resident outcomes. This review mapped the available literature on implementing nurse and caregiver staffing models in LTCFs, provided insight into their implementation, and contributed to the development of a framework to inform staffing models in resource-constrained contexts for older persons.

## References

[CIT0001] Aboderin, I., 2019, ‘Toward a fit-for-purpose policy architecture on long-term care in sub-Saharan Africa: Impasse and a research agenda to overcome it’, *Journal of Long-Term Care* 2019, 119–126. 10.31389/jltc.5

[CIT0002] Al-Jumaili, A.A. & Doucette, W.R., 2018, ‘A systems approach to identify factors influencing adverse drug events in nursing homes’, *Journal of American Geriatrics Society* 66(7), 1420–1427. 10.1111/jgs.15389 29691843

[CIT0003] Aromataris, E., Lockwood, C., Porritt, K., Pilla, B. & Jordan, Z. (eds.), 2024, *JBI manual for evidence synthesis*, JBI, viewed 03 February 2025, from https://jbi-global-wiki.refined.site/space/MANUAL.

[CIT0004] Backhaus, R., Verbeek, H., Van Rossum, E., Capezuti, E. & Hamers, J.P.H., 2014, ‘Nurse staffing impact on quality of care in nursing homes: A systematic review of longitudinal studies’, *Journal of the American Medical Directors Association* 15(6), 383–393. 10.1016/j.jamda.2013.12.080 24529872

[CIT0005] Bae, S.-H. & Kim, H., 2020, ‘Level of resident care need and staffing by size of nursing home under the public long-term care insurance in South Korea’, *Journal of Korean Gerontological Nursing* 22(1), 1–9. 10.17079/jkgn.2020.22.1.1

[CIT0006] Batten, J. & Brackett, A., 2021, ‘Ensuring the rigor in systematic reviews: Part 3, the value of the search’, *Heart & Lung: The Journal of Critical Care* 50(2), 220–222. 10.1016/j.hrtlng.2020.08.005 33340823

[CIT0007] Beckett, C.D., Zadvinskis, I.M., Dean, J., Iseler, J., Powell, J.M. & Buck-Maxwell, B., 2021, ‘An integrative review of team nursing and delegation: Implications for nurse staffing during COVID-19’, *Worldviews on Evidence-Based Nursing* 18(4), 251–260. 10.1111/wvn.12523 34355844 PMC8450812

[CIT0008] Bhaskar, R., 2008, *A realist theory of science*, Routledge, New York, NY.

[CIT0009] Boscart, V.M., Sidani, S., Poss, J., Davey, M., d’Avernas, J., Brown, P. et al., 2018, ‘The associations between staffing hours and quality of care indicators in long-term care’, *BMC Health Services Research* 18(1), 750. 10.1186/s12913-018-3552-5 30285716 PMC6171224

[CIT0010] Brennan, C.W. & Daly, B.J., 2009, ‘Patient acuity: A concept analysis’, *Journal of Advanced Nursing* 65(5), 1114–1126. 10.1111/j.1365-2648.2008.04920.x 19228243

[CIT0011] Brühl, A., Planer, K. & Hagel, A., 2018, ‘Variation of care time between nursing units in classification-based nurse-to-resident ratios: A multilevel analysis’, *INQUIRY* 55, 1–9. 10.1177/0046958018755242 PMC5815415 29442533

[CIT0012] Butler, M., Schultz, T.J., Halligan, P., Sheridan, A., Kinsman, L., Rotter, T. et al., 2019, ‘Hospital nurse-staffing models and patient- and staff-related outcomes’, *Cochrane Database of Systematic Reviews* 4(4), CD007019. 10.1002/14651858.CD007019.pub3 31012954 PMC6478038

[CIT0013] Chappell, V., Kirkham, J. & Seitz, D.P., 2022, ‘Association between long-term care facility staffing levels and antipsychotic use in US long-term care facilities’, *Journal of the American Medical Directors Association* 23(11), 1787–1792. 10.1016/j.jamda.2022.06.029 35926573

[CIT0014] Cho, E., Kim, I., Lee, T., Kim, G., Lee, H. & Min, D., 2020, ‘Effects of registered nurse staffing on quality of care and resident outcomes in nursing homes’, *Geriatric Nursing* 41(6), 685–691. 10.1016/j.gerinurse.2020.04.001 32386999

[CIT0015] Clemens, S., Wodchis, W., McGilton, K., McGrail, K. & McMahon, M., 2021, ‘The relationship between quality and staffing in long-term care: A systematic review of the literature 2008–2020’, *International Journal of Nursing Studies* 122, 104036. 10.1016/j.ijnurstu.2021.104036 34419730

[CIT0016] Dwyer, S.C., 2009, ‘On being an insider-outsider in qualitative research’, *International Journal of Qualitative Methods* 8(1), 54–63. 10.1177/160940690900800105

[CIT0017] Dyer, S.M., Valeri, M., Arora, N., Winsall, M., Tilden, D. & Crotty, M., 2019, *Review of international systems for long-term care of older people*, Flinders University, Adelaide.

[CIT0018] Estabrooks, C.A., Straus, S.E., Flood, C.M., Keefe, J., Armstrong, P., Donner, G.J. et al., 2020, ‘Restoring trust: COVID-19 and the future of long-term care in Canada’, *FACETS* 5(1), 651–619. 10.1139/facets-2020-0056

[CIT0019] Gorges, R.J. & Konetzka, R.T., 2020, ‘Staffing levels and COVID-19 cases and outbreaks in U.S. nursing homes’, *Journal of the American Geriatrics Society* 68(11), 2462–2466. 10.1111/jgs.16787 32770832 PMC7436613

[CIT0020] Griffiths, P., Recio-Saucedo, A., Dall’Ora, C., Briggs, J., Maruotti, A., Meredith, P. et al., 2018, ‘The association between nurse staffing and omissions in nursing care: A systematic review’, *Journal of Advanced Nursing* 74(7), 1474–1487. 10.1111/jan.13564 29517813 PMC6033178

[CIT0021] Griffiths, P., Saville, C., Ball, J.E., Jones, J., Monks, T. & Safer Nursing Care Tool Study Team, 2021, ‘Beyond ratios – Flexible and resilient nurse staffing options to deliver cost-effective hospital care and address staff shortages: A simulation and economic modelling study’, *International Journal of Nursing Studies* 117, 103901. 10.1016/j.ijnurstu.2021.103901 33677251 PMC8220646

[CIT0022] Hamel, C., Garritty, C., Hersi, M., Butler, C., Esmaeilisaraji, L., Rice, D. et al., 2021, ‘Models of provider care in long-term care: A rapid scoping review’, *PLoS One* 16(7), e0254527. 10.1371/journal.pone.0254527 34270578 PMC8284811

[CIT0023] Harrington, C., Choiniere, J., Goldmann, M., Jacobsen, F., Lloyd, L., McGregor, M. et al., 2012, ‘Nursing home staffing standards and staffing levels in six countries’, *Journal of Nursing Scholarship* 44(1), 88–98. 10.1111/j.1547-5069.2011.01430.x 22340814

[CIT0024] Harrington, C., Dellefield, M.E., Halifax, E., Fleming, M.L. & Bakerjian, D., 2020, ‘Appropriate nurse staffing levels for U.S. nursing homes’, *Health Services Insights* 13, 1–14. 10.1177/1178632920934785 PMC7328494 32655278

[CIT0025] Joanna Briggs Institute, 2015, *Joanna Briggs Institute reviewers’ manual 2015. Methodology for JBI scoping reviews*, Joanna Briggs Institute, Adelaide.

[CIT0026] Jutkowitz, E., Landsteiner, A., Ratner, E., Shippee, T., Madrigal, C., Ullman, K. et al., 2023, ‘Effects of nurse staffing on resident outcomes in nursing homes: A systematic review’, *Journal of the American Medical Directors Association* 24(1), 75–81. 10.1016/j.jamda.2022.11.002 36470321

[CIT0027] Juvé-Udina, M., Adamuz, J., López-Jimenez, M., Tapia-Pérez, M., Fabrellas, N., Matud-Calvo, C. et al., 2019, ‘Predicting patient acuity according to their main problem’, *Journal of Nursing Management* 27(8), 1845–1858. 10.1111/jonm.12885 31584733 PMC7328732

[CIT0028] Kim, Y. & Han, K., 2018, ‘Longitudinal associations of nursing staff turnover with patient outcomes in long-term care hospitals in Korea’, *Journal of Nursing Management* 26(5), 518–524. 10.1111/jonm.12576 29318685

[CIT0029] Koopmans, L., Damen, N. & Wagner, C., 2018, ‘Does diverse staff and skill mix of teams impact quality of care in long-term elderly health care? An exploratory case study’, *BMC Health Services Research* 18(1), 988. 10.1186/s12913-018-3812-4 30572880 PMC6302304

[CIT0030] Lee, H.Y., Shin, J.H. & Harrington, C.A., 2015, ‘Comparing the nurse staffing in Korean and U.S. nursing homes’, *Nursing Outlook* 63(2), 137–143. 10.1016/j.outlook.2014.08.005 25261384

[CIT0031] Lee, J., Shin, J.H., Lee, K.H., Harrington, C.A. & Jung, S.O., 2022, ‘Staffing levels and COVID-19 infections and deaths in Korean nursing homes’, *Policy, Politics & Nursing Practice* 23(1), 15–25. 10.1177/15271544211056051 PMC8801339 34939511

[CIT0032] Lerner, N.B., 2013, ‘The relationship between nursing staff levels, skill mix, and deficiencies in Maryland nursing homes’, *The Health Care Manager* 32(2), 123–128. 10.1097/HCM.0b013e31828ef5f9 23629034

[CIT0033] Lincoln, Y.S. & Guba, E.G., 1985, *Naturalistic inquiry*, Sage Publications Inc., California, CA.

[CIT0034] Madungwe, L.S., Mupfumira, I.M. & Chindedza, W., 2011, ‘A comparative study of the culture of skilled nursing facilities in high and low density areas: A case for Masvingo urban in Zimbabwe’, *Journal of Sustainable Development in Africa* 13(1), 1–12.

[CIT0035] McCloskey, R., Donovan, C., Stewart, C. & Donovan, A., 2015, ‘How registered nurses, licensed practical nurses and resident aides spend time in nursing homes: An observational study’, *International Journal of Nursing Studies* 52(9), 1475–1483. 10.1016/j.ijnurstu.2015.05.007 26117710

[CIT0036] Mlinac, M. & Feng, M.C., 2016, ‘Assessment of activities of daily living, self-care, and independence’, *Archives of Clinical Neuropsychology* 31, 506–516. 10.1093/arclin/acw049 27475282

[CIT0037] Mueller, C., 2000, ‘A framework for nurse staffing in long-term care facilities’, *Geriatric Nursing* 21(5), 262–267. 10.1067/mgn.2000.110834 11035310

[CIT0038] Mukamel, D.B., Saliba, D., Ladd, H. & Konetzka, R.T., 2023, ‘Association of staffing instability with quality of nursing home care’, *JAMA Network Open* 6(1), e2250389. 10.1001/jamanetworkopen.2022.50389 36626170 PMC9856742

[CIT0039] Nicholson, E.C., Van der Heever, M.M., Young, C. & Van der Merwe, A., 2024, ‘Developing a framework to inform staffing models for long-term care facilities in resource-constrained contexts’, PhD thesis, Stellenbosch University, Stellenbosch.

[CIT0040] Page, B., Irving, D., Amalberti, R. & Vincent, C., 2023, ‘Health services under pressure: A scoping review and development of a taxonomy of adaptive strategies’, *BMJ Quality & Safety* 33(11), 738–747. 10.1136/bmjqs-2023-016686 PMC11503202 38050158

[CIT0041] Perruchoud, E., Weissbrodt, R., Verloo, H., Fournier, C.-A., Genolet, A., Rosselet Amoussou, J. et al., 2021, ‘The impact of nursing staffs’ working conditions on the quality of care received by older adults in long-term residential care facilities: A systematic review of interventional and observational studies’, *Geriatrics (Basel, Switzerland)* 7(1), 6. 10.3390/geriatrics7010006 35076476 PMC8788263

[CIT0042] Peters, M.D.J., Marnie, C., Tricco, A.C., Pollock, D., Munn, Z., Alexander, L. et al., 2020, ‘Updated methodological guidance for the conduct of scoping reviews’, *JBI Evidence Synthesis* 18(10), 2119–2126. 10.11124/JBIES-20-00167 33038124

[CIT0043] Republic of South Africa, 2005, *Nursing Act 33 of 2005*, Government Printer, Pretoria.

[CIT0044] Republic of South Africa, 2006, *Older Persons Act 13 of 2006*, Government Printer, Pretoria.

[CIT0045] Republic of South Africa, 2010a, *Regulations regarding older persons*, Government Gazette no. 33075, 01 April, Government Printing Works, Pretoria.

[CIT0046] Republic of South Africa, 2010b, *Final report. Audit of residential facilities*, Department of Social Development, Pretoria.

[CIT0047] Republic of South Africa, 2015, *Health standards/norms for residential facilities for older persons*, Western Cape Government, Cape Town.

[CIT0048] Republic of South Africa, 2022, *Regulations regarding the scope of practice for nurses and midwifes*, Government Gazette no. 46471, 03 June, Government Printing Works, Pretoria.

[CIT0049] Sato, N., Akazawa, K., Mitadera, Y., Suzuki, T., Ibe, N. & Hirose, Y., 2017, ‘Clarifying problems with emergency healthcare systems in Japanese long-term care facilities for older people’, *Health* 9(8), 1159–1175. 10.4236/health.2017.98084

[CIT0050] Schnelle, J.F., Schroyer, L.D., Saraf, A.A. & Simmons, S.F., 2016, ‘Determining nurse aide staffing requirements to provide care based on resident workload: A discrete event simulation model’, *Journal of American Medical Directors Association* 17(11), 970–977. 10.1016/j.jamda.2016.08.006 27780572

[CIT0051] Shin, J.H., 2013, ‘Relationship between nursing staffing and quality of life in nursing homes’, *Contemporary Nurse* 44(2), 133–143. 10.5172/conu.2013.44.2.133 23869498

[CIT0052] Shin, J.H., 2018, ‘Why do we require registered nurses in nursing homes? Using longitudinal hierarchical linear modelling’, *Journal of Nursing Scholarship: An Official Publication of Sigma Theta Tau International Honor Society of Nursing* 50(6), 705–713. 30043547 10.1111/jnu.12412

[CIT0053] Shin, J.H., 2019, ‘Appropriate nursing home nurse hours per resident day in Korea: A secondary analysis of longitudinal data’, *Journal of Nursing Scholarship* 51(5), 569–579. 31328880 10.1111/jnu.12498

[CIT0054] Shin, J.H. & Hyun, T.K., 2015, ‘Nurse staffing and quality of care of nursing home residents in Korea’, *Journal of Nursing Scholarship* 47(6), 555–564. 10.1111/jnu.12166 26467903

[CIT0055] Shin, J.H., Park, T. & Huh, I., 2014, ‘Nursing staffing and quality of life in Western New York Nursing Homes’, *Western Journal of Nursing Research* 36(6), 788–805. 10.1177/0193945913511154 24258404

[CIT0056] Shin, J.H., Renaut, R.A., Reiser, M., Lee, J.Y. & Tang, T.Y., 2021, ‘Increasing registered nurse hours per resident day for improved nursing home residents’ outcomes using a longitudinal study’, *International Journal of Environmental Research and Public Health* 18(2), 402. 10.3390/ijerph18020402 33419183 PMC7825529

[CIT0057] Shin, J.H. & Shin, I.-S., 2019, ‘The effect of registered nurses on nursing home residents’ outcomes, controlling for organizational and health care market factors’, *Geriatric Nursing* 40(3), 296–301. 10.1016/j.gerinurse.2018.11.004 30528039

[CIT0058] South African Nursing Council, 1984, *Regulations relating to the scope of practice of persons who are registered or enrolled under the Nursing Act, 1978*, South African Nursing Council, Pretoria.

[CIT0059] South African Qualifications Authority, 2022, *National qualifications framework*, viewed 15 April 2023, from https://www.saqa.org.za/.

[CIT0060] Tuinman, A., De Greef, M.H.G., Krijnen, W.P., Nieweg, R.M.B. & Roodbol, P.F., 2016, ‘Examining time use of Dutch nursing staff in long-term institutional care: A time-motion study’, *Journal of the American Medical Directors Association* 17(2), 148–154. 10.1016/j.jamda.2015.09.002 26482057

[CIT0061] United Nations, 2022, *World population prospects 2022*, United Nations Publication, New York, NY.

[CIT0062] Van Eeuwijk, P., 2014, ‘The elderly providing care for the elderly in Tanzania and Indonesia: Making “elder to elder” care visible’, *Sociologus* 64(1), 29–52. 10.3790/soc.64.1.29

[CIT0063] World Health Organization (WHO), 2015, *World report on ageing and health*, WHO Press, Luxembourg.

[CIT0064] World Health Organization (WHO), 2017, *Towards long-term care systems in sub-Saharan Africa. WHO series on long-term care on healthy ageing*, WHO Document Production Services, Geneva.

[CIT0065] World Health Organization (WHO), 2022, *Nursing and midwifery*, viewed 17 November 2023, from https://www-who-int.ez.sun.ac.za/news-room/fact-sheets/detail/nursing-and-midwifery.

[CIT0066] Yang, B.K., Carter, M.W., Trinkoff, A.M. & Nelson, H.W., 2021, ‘Nurse staffing and skill mix patterns in relation to resident care outcomes in US nursing homes’, *Journal of the American Medical Directors Association* 22(5), 1081–1087.e1. 10.1016/j.jamda.2020.09.009 33132015

[CIT0067] Yoon, J.M., Trinkoff, A.M., Galik, E., Storr, C.L., Lerner, N.B., Brandt, N. et al., 2022, ‘Nurse staffing and deficiency of care for inappropriate psychotropic medication use in nursing home residents with dementia’, *Journal of Nursing Scholarship* 54(6), 728–737. 10.1111/jnu.12776 35388951

[CIT0068] Zimmermann, J. & Pfaff, H., 2018, ‘Influence of nurse staffing levels on resident weight loss within German nursing homes’, *Research in Gerontological Nursing* 11(1), 48–56. 10.3928/19404921-20180109-01 29370446

[CIT0069] Zirves, M., Demirer, I. & Pfaff, H., 2021, ‘Everyday life and social contacts of dementia and non-dementia residents over 80 years in long-term inpatient care: A multi-level analysis on the effect of staffing’, *International Journal of Environmental Research and Public Health* 18(21), 11300. 10.3390/ijerph182111300 34769817 PMC8583643

